# Porcelain aorta does not mean inoperability but needs special strategies

**DOI:** 10.1093/icvts/ivac222

**Published:** 2022-08-22

**Authors:** Thierry Carrel, Paul Robert Vogt

**Affiliations:** Department of Cardiac Surgery, University Hospital Zürich, Zürich, Switzerland; Department of Cardiac Surgery, University Hospital Zürich, Zürich, Switzerland

**Keywords:** Porcelain aorta, Aortic valve replacement, Coronary bypass grafting, Xeno-pericardial patch, Hypothermic circulatory arrest

## Abstract

Porcelain aorta is not an absolute contraindication for aortic valve and/or coronary bypass grafting but it requires a special strategy and individualized approach to minimize the risk of embolic complications and technical problems during opening and/or closing the aortotomy.

The overall incidence of significant calcification of the ascending aorta is reported in 5–8% of patients scheduled for aortic valve replacement (AVR) and/or coronary artery bypass grafting (CABG) [1]. Porcelain aorta is usually considered as a contraindication to conventional surgery because every aortic manipulation may imply a risk of calcific embolization that results in neurological complications [2]. For this reason, catheter-based procedures are largely recommended [3]. However, we believe that multiple strategies are still available to minimize the risk of embolization of calcific aortic debris during a cardiosurgical procedure.

## CASES AND OPERATIVE TECHNIQUE

We present 2 cases in which, depending on the location and extent of calcifications, an individual strategy was applied.

**Figure 1:**
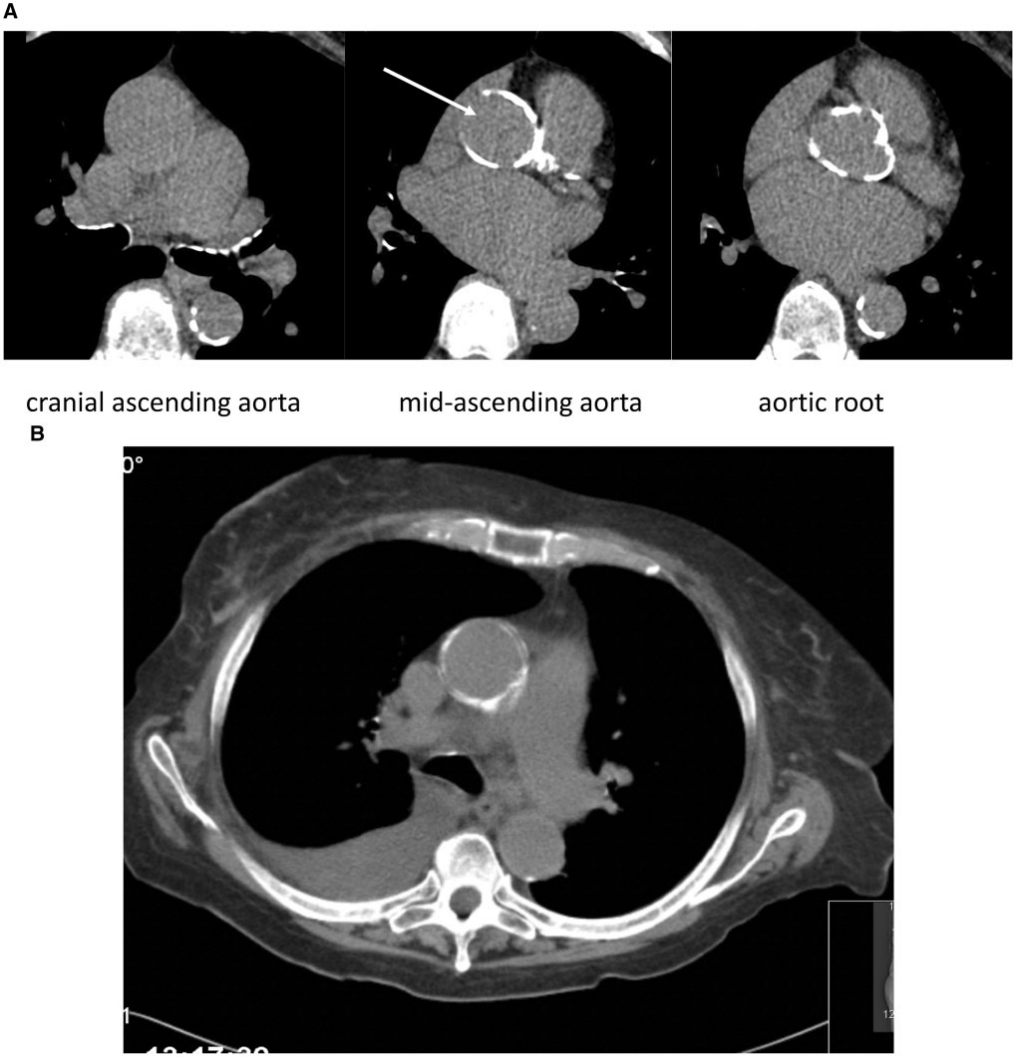
(**A**) Computed tomography showing the calcifications at different level of the ascending aorta and the aortic root with the ‘calcium-free window’ on the right lateral wall (white arrow), where the aorta was opened for aortic valve replacement. (**B**) Coronal view of the ascending aorta.

A 72-year-old female was scheduled for combined AVR and CABG because of a severe aortic stenosis [mean/max pressure gradient was 55/80 mmHg, and valve opening area was 0.8 cm^2^ (indexed 0.55 cm^2^) with a narrowed aortic annulus of 18 mm and a subaortic stenosis]. In addition, angiography showed significant stenosis of the left main coronary artery. Preoperative computed tomography scan demonstrated important calcifications of the proximal two-thirds of the ascending aorta and the aortic root, with exception of a small island in the lateral wall (Fig. 1). Because of the subaortic stenosis, the small aortic annulus and the left main stenosis, conventional surgery was recommended.

**Figure 2:**
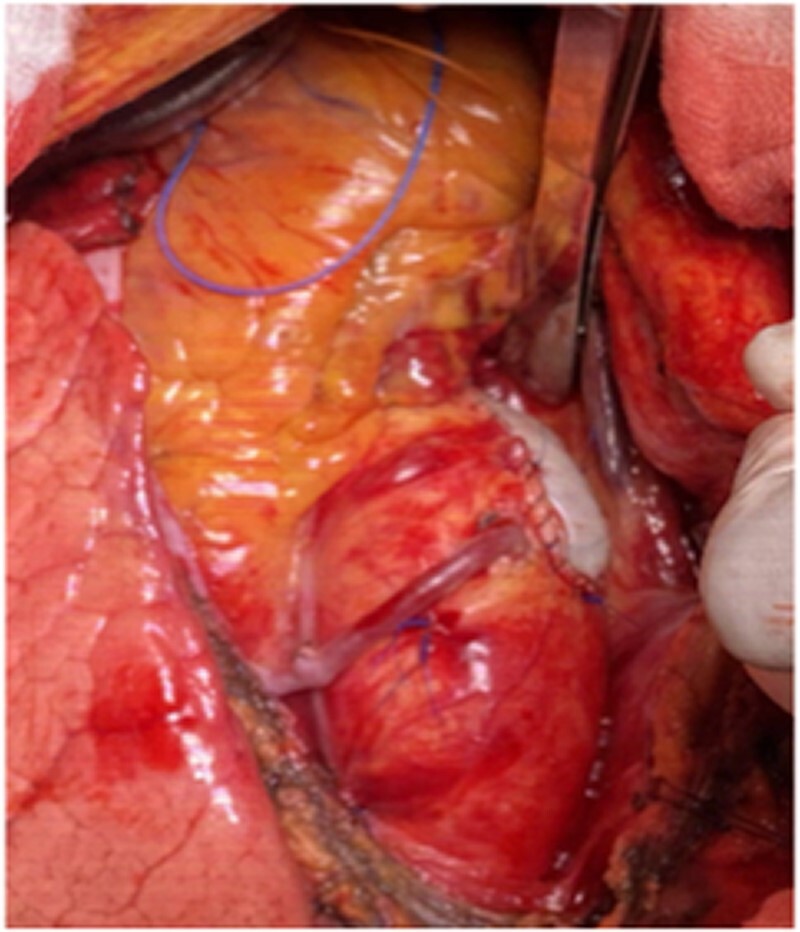
Intraoperative view following closure of the aortotomy with the same xeno-pericardial patch used for annulus enlargement and proximal anastomosis of the saphenous vein graft close to the patch in the non-calcified aortic wall.

Surgery was performed through median sternotomy and aortic cannulation was done in the proximal aortic arch. The aorta was clamped very cranially just proximally to the innominate artery. Because of the extension of calcifications, a longitudinal instead of a transverse aortotomy was performed on the right lateral side of the ascending aorta. Endarterectomy was considered as too risky. The incision extended up into the commissure between the left and non-coronary cusps and further down into the anterior leaflet of the mitral valve to enlarge the aortic annulus. Subaortic resection was done according to Morrow's technique. A xeno-pericardial patch was attached to the anterior leaflet of the mitral valve and used later in its full length to close the lateral aortotomy (Fig. 2). A 21-mm tissue valve (Inspiris, Edwards, CA, USA) was implanted using Ethibound 2.0 pledgeted non-everting sutures. Double coronary bypass using the left internal thoracic artery for the left descending branch and a saphenous vein graft for the marginal branch was performed concomitantly (the proximal vein graft anastomosis being performed during cardiac arrest close to the patch to avoid additional any further aortic clamping). Cardiopulmonary bypass time was 98 min with a myocardial ischaemia of 69 min. Postoperative recovery was uneventful and the patient could be discharged on postoperative day 8.

The second patient was a 78-year-old female suffering from a severe aortic stenosis that had presented recurrent transient ischaemic attacks. She had no atrial fibrillation nor a patent foramen ovale but was found to have circular and mobile calcifications of the ascending aorta, precluding a safe clamping. She underwent AVR and replacement of the ascending aorta in moderate hypothermia and a short period of circulatory arrest (12 min) for the distal anastomosis. This strategy allowed us to avoid aortic clamping and resulted in an uncomplicated postoperative outcome.

Both patients gave informed consent for the procedure and for the presentation of the data.

## COMMENT

The recent guidelines of the European Society of Cardiology/European Association for Cardio-Thoracic Surgery (ESC/EACTS) consider the porcelain aorta as a condition in which catheter-based procedures should be preferred [3]. Although this statement is justified for older and highest-risk patients, a conventional surgical approach including an individualized technique to minimize the risk of embolization may be justified and feasible with reasonable results. There are numerous surgical strategies to avoid or minimize the risk of particle embolization when dealing with a porcelain aorta:

1. peripheral cannulation site for arterial return,
2. intraluminal balloon occlusion of the aorta,
3. aortic replacement during hypothermic circulatory arrest to avoid cross-clamping [4],
4. limited or extended aortic endarterectomy [5],
5. slow aortic clamping with flushing through the open aorta [6]
6. no aortic touch off-pump coronary bypass grafting using *in situ* T or Y arterial grafts [7]
7. sutureless AVR to speed up surgery if the latter is performed during circulatory arrest and to minimize manipulations within a heavily calcified aortic root [8].

In a previous paper, we had collected data on 52 consecutive patients with a porcelain aorta who underwent a conventional cardiosurgical procedure [9]. Alternative cannulation sites were used in 40 patients. In a majority of patients, the ascending aorta was replaced with the distal anastomosis performed during hypothermic circulatory arrest (±local endarterectomy at the anastomotic site), while the cardiac part of the procedure (AVR, CABG) was performed during rewarming with the prosthetic graft clamped.

In the remaining patients, CABG was performed on the beating heart with T- or Y-grafts using both internal thoracic arteries as the unique blood source for the revascularization. Before the transcatheter valve era, we performed AVR as an apico-aortic conduit from a lateral thoracotomy under peripheral cardiopulmonary bypass and ventricular fibrillation in 3 patients. The results were promising with an overall neurological complication rate of 9.6% (5/52); 3 of these complications resolved completely until the patient was discharged.

Dealing with a porcelain aorta during a cardiac surgery is not something new but it merits to be emphasized in the era of transcatheter technologies. Younger cardiac surgeons should be aware that porcelain aorta does not mean inoperability but that an individualized approach may be offered with a reasonable risk of neurological complications, especially in case transcatheter aortic valve replacement (TAVR) and/or percutaneous coronary intervention (PCI) is not indicated nor feasible.

**Conflict of interest:** none declared.

## Reviewer information

Interactive CardioVascular and Thoracic Surgery thanks Dimitrios C. Angouras and the other anonymous reviewer(s) for their contribution to the peer review process of this article.

## Notes

The need for informed written consent was waived by the IRB/ERB and the patient gave informed consent for the procedure itself and for the presentation of these data.
